# Necroptosis‐related regulatory pattern and scoring system for predicting therapeutic efficacy and prognosis in ovarian cancer

**DOI:** 10.1002/cnr2.1893

**Published:** 2023-09-08

**Authors:** Huiling Lai, Yunyun Guo, Linxiang Wu, Aligu Yusufu, Qiyu Zhong, Zhouzhou Liao, Jianyu Ma, Wen Shi, Guofen Yang, Shuqin Chen

**Affiliations:** ^1^ Department of Gynecology, The Sixth Affiliated Hospital Sun Yat‐Sen University Guangzhou China; ^2^ Center of Basic Medical Research, Institute of Medical Innovation and Research Peking University Third Hospital Beijing China; ^3^ Department of Gynecology, The First Affiliated Hospital Sun Yat‐Sen University Guangzhou China

**Keywords:** biomarkers, clustering analysis, necroptosis, ovarian cancer

## Abstract

**Background:**

Ovarian cancer is difficult to treat and is, therefore, associated with a high fatality rate. Although targeted therapy and immunotherapy have been successfully used clinically to improve the diagnosis and treatment of ovarian cancer, most tumors become drug resistant, and patients experience relapse, meaning that the overall survival rate remains low.

**Aims:**

There is currently a lack of effective biomarkers for predicting the prognosis and/or outcomes of patients with ovarian cancer. Therefore, we used published transcriptomic data derived from a large ovarian cancer sample set to establish a molecular subtyping model of the core genes involved in necroptosis in ovarian cancer.

**Methods and Results:**

Clustering analysis and differential gene expression analyses were performed to establish the genomic subtypes related to necroptosis and to explore the patterns of regulatory gene expression related to necroptosis in ovarian cancer. A necroptosis scoring system (NSS) was established using principal component analysis according to different regulatory patterns of necroptosis. In addition, this study revealed important biological processes with essential roles in the regulation of ovarian tumorigenesis, including external encapsulating structure organization, leukocyte migration, oxidative phosphorylation, and focal adhesion. Patients with high NSS scores had unique immunophenotypes, such as more abundant M2 macrophages, monocytes, CD4^+^ memory T cells, and regulatory T cells. Immune checkpoint CD274 had a greater expression in patients with high NSS values.

**Conclusion:**

This NSS could be used as an independent predictor of prognosis to determine the sensitivity of ovarian cancer to various small‐molecule inhibitors, immune checkpoint inhibitors, and platinum‐based chemotherapy drugs.

## 1 INTRODUCTION

Although ovarian cancer accounts for only 2.5% of all malignancies among females, the high fatality rate associated with it is responsible for 5% of cancer‐related deaths.^1^ Owing to the lack of typical symptoms and effective screening methods, the percentage of patients with advanced‐stage disease (Federation of Gynecology and Obstetrics [FIGO] stage III or IV) exceeds 70%, and the 5‐year survival rate of patients with ovarian cancer is approximately 46%.^2^

Ovarian cancer is also difficult to treat because of the high rates of relapse, and relapsed tumor cells gradually become drug resistant.^3^ Great advances have been made in the past few decades for the treatment of ovarian cancer, including the successful clinical application of poly ADP‐ribose polymerase inhibitor (PARPi); however, resistance to PARPi remains a challenge.^4^ In addition, there are no reliable biomarkers or predictive models to guide the selection and application of drugs to treat recurrent ovarian cancer, and second‐ or third‐line regimens can only be selected based on evidence‐based medical data. Consequently, many patients have no effective therapeutic options or die from drug‐related adverse reactions.^5^

Although cancer immunotherapies using immune checkpoint inhibitors (ICIs) have achieved remarkable success in clinical practice,^6^ only a third of patients with cancer respond to ICIs, and hence, the number of patients who can benefit is limited. ICI therapy is also limited by the occurrence of immune‐related adverse events caused by immune overactivation and the subsequent disturbance of immune homeostasis.^7^ Indeed, serious adverse events can lead to permanent illness, and, in some cases, death. Therefore, it is important to develop predictive and prognostic biomarkers for ICI therapies to better understand their benefits and risks and to effectively select patients for these therapies.

Inducing cell death has become a promising novel cancer treatment strategy since most tumors display innate resistance to apoptosis.^8^ Several recent studies have revealed the interactions between different cell death mechanisms and antitumor immunity. For instance, the combinatorial application of ICIs and drugs that induce pyroptosis and ferroptosis exerts enhanced synergistic antitumor activity, even in ICI‐resistant tumors.^9^ Necroptosis is a form of regulated cell death that has the same morphological features as necrosis.^10^ Receptor‐interacting serine–threonine kinase 3 (RIPK3) and mixed lineage kinase domain‐like (MLKL) play important roles in necroptosis, and recent studies have begun to elucidate the detailed molecular mechanisms underlying necroptosis under different pathophysiological conditions.^11^ In particular, key components of necroptosis appear to be downregulated in tumors,^12^ and accumulating evidence has indicated that necroptosis‐deficient cancer cells are poorly immunogenic, and hence escape natural and therapy‐elicited immunosurveillance. Unfortunately, the role of necroptosis in ovarian cancer development remains unclear.

This research aimed to elucidate the role of necroptosis in the pathogenesis of ovarian cancer, facilitate the development of new molecular typing methods, guide treatment options, and effectively select patients who may benefit from immunotherapy.

## 2 METHODS

### 2.1 Data preparation and preprocessing

**FIGURE 1:**
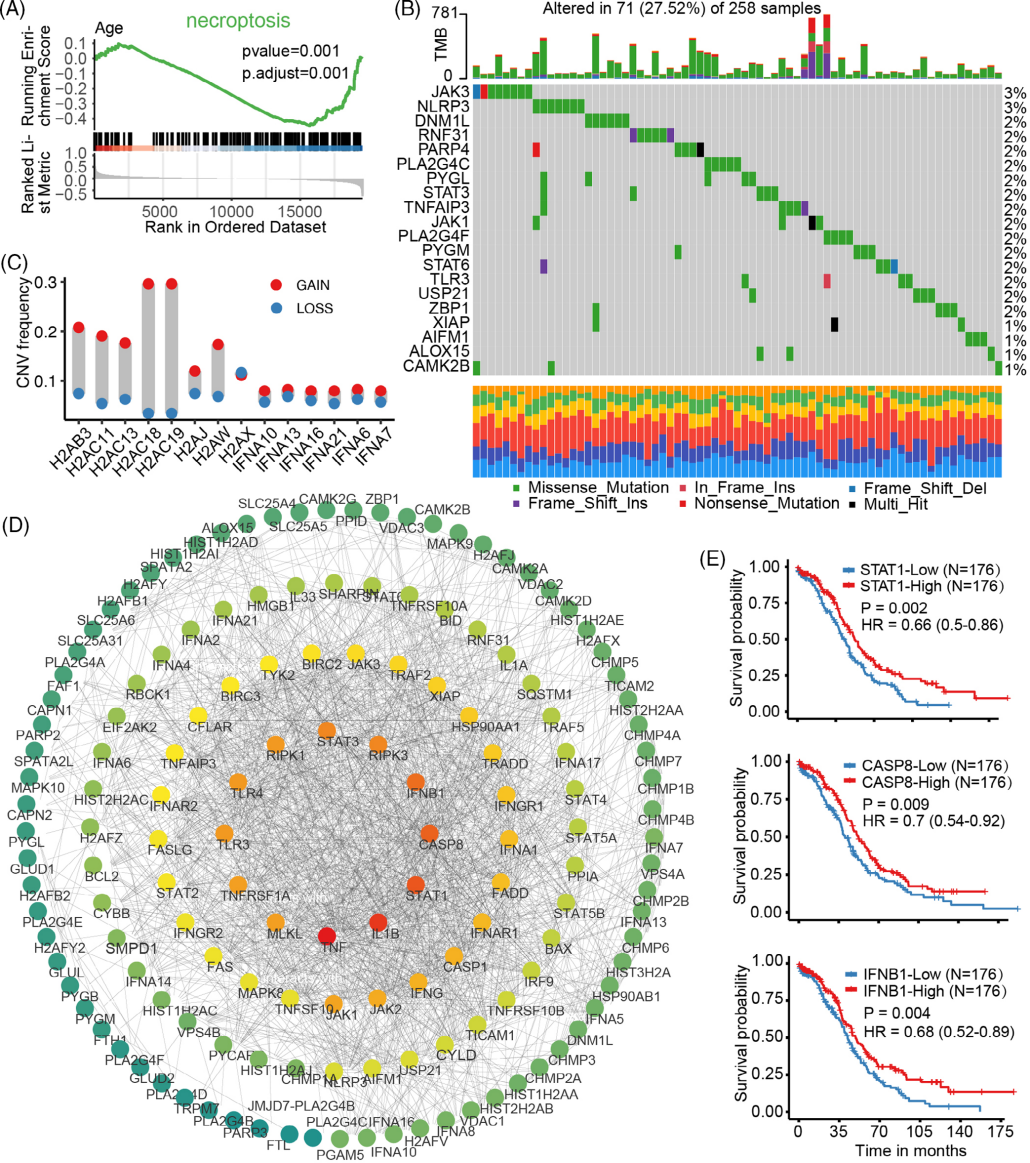
Necroptosis affects the development of ovarian cancer. (A) Gene enrichment analysis of necroptosis‐related genes in different age groups. (B) Top 20 mutation frequencies in the TCGA‐OV cohort. Different columns represent individual patients. The bar chart above shows the tumor mutation burden (TMB). The numbers on the right indicate the mutation frequency of each gene. The stack bar chart below shows the conversion rates in each sample. (C) Copy number variation (CNV) of necroptosis‐related genes. The ordinate refers to the frequency of CNV variation (that is, the proportion of samples with copy number amplification or loss). The different genes are listed on the horizontal axis. Red dots represent copy number amplification and blue dots represent copy number loss. (D) Interaction network of necroptosis‐related genes. (E) Survival analysis of core genes in the interaction network.

**FIGURE 2:**
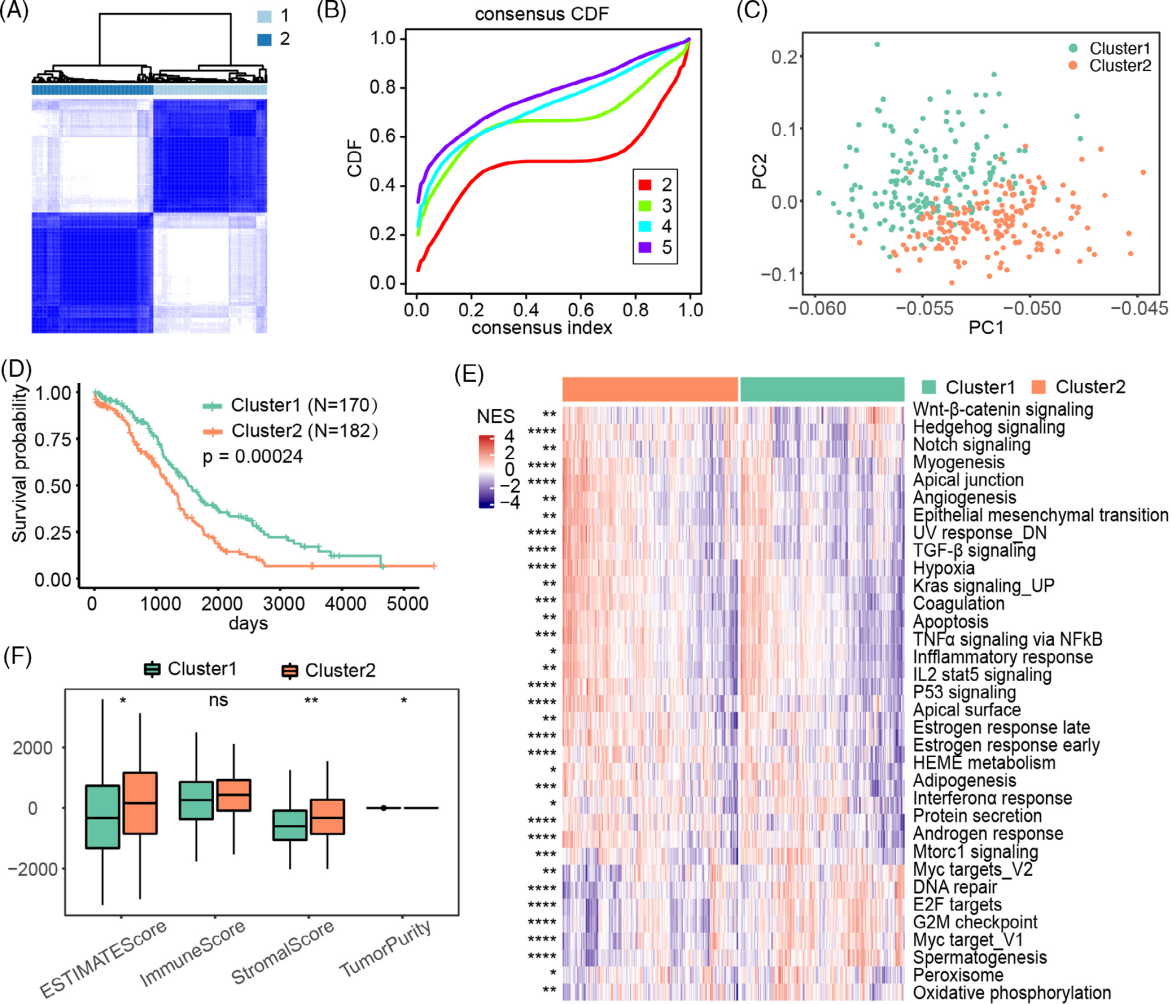
Identification of different ovarian cancer subtypes based on necroptotic genes. (A) Consistent clustering based on the expression of 15 core necroptosis‐related genes in the TCGA‐OV cohort. (B) Distribution of CDF curves in consistent clustering. (C) PCA analysis of the two subtypes. (D) Survival status of different subtypes. (E) Comparison of HALLMARK pathways in different subtypes. (F) Comparison of ESTIMATE score, immune score, stromal score, and tumor purity between the two subtypes.

Clinical information and transcriptomic (log2(FPKM+1)) data for ovarian cancer (OV) samples were acquired from The Cancer Genome Atlas (TCGA) database using the R package TCGAbiolinks. The expression and survival information of 352 tumor samples from TCGA‐OV dataset, which included no normal samples, was used for subsequent analyses. Because the control group for a specific comparative analysis is different, the control and experimental groups in each comparative analysis are described in detail in the part “Result” or figures. For example, in Section 3.1 (the experimental group and control group for comparison were also marked in Figure 1A), the the control group refer to patients ≥60 years old. In Section 3.2 (the experimental group and control group for comparison were also marked in Figure 2D), the control group are Cluster2.

The prediction model was verified using the GSE132342 dataset from the Gene Expression Omnibus (GEO) database. The expression data of this dataset were determined using the GPL26748 platform.

Validation cohort data were processed as follows: (1) deletion of no‐load probe; (2) deletion of probes corresponding to multiple genes; (3) calculation of median expression values for multiple probes corresponding to the same gene.

Map0421 necroptosis pathways in *Homo sapiens* were selected from the Kyoto Encyclopedia of Genes and Genomes (KEGG, https://www.kegg.jp) website. In total, 160 necroptosis‐associated genes were extracted after ENTREZID transformation.

### 2.2 Gene set enrichment analysis (GSEA), copy number variation (CNV), genetic mutations, and survival analysis of necroptosis‐associated genes

GSEA was used to calculate whether selected gene sets differed significantly between the experimental and control groups. The enrichment of necroptosis‐associated genes in patients with different clinical features was analyzed using the R “clusterProfiler” package. CNVs are copy number amplifications and deletions in genomic regions larger than 1 Kilobase (kb). The frequency of CNV refers to the proportion of the number of samples in which CNV occurs to the total sample. For example, the frequency of GAIN for H2AC18 gene was calculated as follow: 76 (samples with copy number amplification in genomic regions larger than 1 kb)/258 (samples with CNV data available for analysis) = 0.2946. The frequency of LOSS for H2AC18 gene was calculated as follow: 8 (samples with copy number deletion in genomic regions larger than 1 kb)/258 = 0.031. A CNV was considered common if its population frequency is >1%. The “maftools” package was utilized to analyze and visualize mutations in necroptosis‐associated genes in TCGA‐OV samples.

### 2.3 Protein–protein interaction (PPI) network analysis

The interactions among necroptosis‐associated genes were investigated in the STRING database. A PPI network was generated, and a network diagram was generated using Cytoscape (v.3.9.1).

### 2.4 Clustering of ovarian cancer subtypes

Univariate Cox regression analysis was performed on the 160 necroptosis‐associated genes using the R “survival” package. Core genes were screened using a threshold of *p* < .05. TCGA‐OV tumor samples were classified based on these core genes. These samples were subjected to consistent clustering analysis using the “ConsensusClusterPlus” package with Euclidean distance and the pam clustering method (reps = 100, pItem = 0.8). The Kaplan–Meier (KM) method was used to generate the prognostic survival curves for different ovarian cancer subtypes. The log‐rank test was utilized to evaluate the significance of differences between these curves.

Gene set variation analysis (GSVA) for different ovarian cancer subtypes was performed using the “msigdb.v7.4. symbols.gmt” gene set, which was extracted from the MSigDB database. Results with *p* < .05 were considered statistically significant.

### 2.5 Gene ontology (GO) enrichment and KEGG pathway analysis of differentially expressed genes (DEGs)

Differential patterns of expression between the two ovarian cancer subtypes were analyzed using the R “limma” package. DEGs were screened according to these parameters: log2(Fold Change, FC) > 0.585 (or < −0.585) and *p* < 0.05. Specially, log2(FC) > 0.585 means up‐regulation, while log2(FC) < −0.585 suggests down‐regulation. The “ClusterProfiler” package was used to conduct KEGG and GO analyses of the DEGs with the following parameters: pvalueCutoff = 0.05, pAdjustMethod = “BH,” and qvalueCutoff = 0.5. The top 10 pathways were visualized.

### 2.6 DEG re‐clustering analysis

TCGA‐OV samples were re‐clustered based on the identified DEGs using the R “ConsensusClusterPlus” package with Euclidean distances and the k‐means clustering algorithm (reps = 100, pItem = 0.8). The R “survival” package was used to analyze survival in the re‐clustered subtypes, and “ggplot2” was used to draw box plots of differences in the expression of necroptosis‐related genes.

### 2.7 Construction of the necroptosis scoring system (NSS)

To quantify necroptosis for individual tumors, we constructed a scoring system named “NSS.” First, univariate Cox regression analysis was performed, aiming to discover the hazard ratio (HR) and prognostic significance of DEGs. Prognosis‐related genes were defined as those with *p* < .01. Next, the NSS was constructed using principal component analysis (PCA) based on the prognosis‐related genes. Principal components 1 and 2 were feature scores. NSS was developed using the genes with the highest correlation (or anti‐correlation) in the gene set, thus reducing the contribution weight of genes with other unrelated members of the gene set. The following formula was used:

(1) $$\mathrm{NSS}=\sum \;\left(\mathrm{PC}1i+\mathrm{PC}2i\right),$$

where *i* represents individual tumor samples.

Ovarian cancer samples were grouped into different groups based on the median values by NSS, and the survival status of patients was analyzed. Univariate and multivariate Cox regression analyses were performed to explore the independent prognostic value of the NSS. The same formula was used to calculate the NSS in the validation dataset.

### 2.8 Immuno‐infiltration analysis

The CIBERSORT algorithm of the “IOBR” package was utilized to calculate immune cell fractions in the tumor microenvironment, with perm = 200. The matrix score, immune score, Estimation of Stromal and Immune cells in Malignant Tumor tissues using Expression (ESTIMATE) score, and tumor purity were calculated using the R “ESTIMATE” package.

### 2.9 Predictive value of NSS for chemotherapy drug sensitivity

The genomics study of cancer drug sensitivity (GDSC) database data were analyzed using the R‐encapsulated oncoPredict to assess the IC_50_ value of a drug in different NSS groups, which can be used to evaluate the sensitivity of an antitumor drug. Spearman's correlation was calculated between the NSS values and the IC_50_ of drugs.

### 2.10 Statistical analysis

All statistical analyses were performed using R (v.4.1.2). To determine the significance of differences in expression levels, infiltration ratios, and various eigenvalues, the Wilcoxon rank‐sum test was used to compare two groups of samples, and the Kruskal–Wallis test was used to compare multiple groups of samples. In all figures, significant differences are indicated as follows: ns = *p* > .05, * = *p* ≤ .05, ** = *p* ≤ .01, *** = *p* ≤ .001, and ^****^ = *p* ≤ .0001. Survival curves were generated using the KM method. The significance of differences was evaluated using the log‐rank test.

## 3 RESULTS

### 3.1 Necroptosis affects the pathophysiology of ovarian cancer

Based on the ovarian cancer transcriptomic data in the TCGA‐OV dataset, we found that necroptosis‐related genes were significantly downregulated in patients with ovarian cancer aged ≥60 years (Figure 1A) and that 27.52% (71/258) of patients harbored genetic mutations, including *JAK3*, *NLRP3*, *DNM1L*, *RNF31*, *PARP4*, *PLA2G4C*, *PYGL*, and so on (Figure 1B). In addition, CNV was common, especially in the *H2AC18* and *H2AC19* genes (Figure 1C). Further exploration of the interactions among the necroptosis‐associated genes using String data revealed that *TNF*, *IL1B*, *STAT1*, *CASP8*, and *IFNB1* had high network connectivity (Figure 1D). In addition, patients with high *STAT1*, *CASP8*, and *IFNB1* expression tended to have a better prognosis, as determined using KM analysis (Figure 1E). Taken together, these findings suggest that necroptosis participates in the pathophysiology of ovarian cancer.

### 3.2 Identification of ovarian cancer subtypes based on necroptosis‐associated genes

Next, we performed univariate Cox regression analysis on the necroptosis‐associated genes. Among the 160 necroptotic genes, 15 core genes were selected with *p* < .05. Based on the expression data for these 15 core genes in TCGA‐OV, consensus clustering was conducted using the R “ConsensusClusterPlus” package (Figure 2A). A cumulative distribution function (CDF) curve was generated, based on which samples were divided into the two subtypes (Figure 2B). The differences between Cluster1 and Cluster2 were determined using PCA (Figure 2C). KM analysis revealed that Cluster1 was associated with a better survival than Cluster2 (Figure 2D). Therefore, we explored the biological behaviors of the different subtypes by using GSVA to evaluate differences in HALLMARK pathways (Figure 2E). Common signaling pathways that were activated in tumors differed significantly between different subtypes, including those related to DNA repair, Hedgehog, TGF‐β, TNF‐α via NF‐κB, Notch, Wnt‐β‐catenin, angiogenesis, Il2‐stat5, and the inflammatory response (Figure 2E). Immune cell infiltration analysis using the CIBERSORT algorithm revealed significant differences between the two subtypes, including M1 macrophages, follicular helper T cells, γδT cells and CD4 memory resting T cells (Figure S2). ESTIMATE data analysis further revealed that patients with a Cluster1 subtype had significantly lower ESTIMATE and matrix scores than those with a Cluster2 subtype (Figure 2F). In general, ovarian cancer could be divided into two subtypes based on differential expression of necroptotic genes.

### 3.3 Differential expression analysis and regulatory patterns of different subtypes based on necroptosis

**FIGURE 3:**
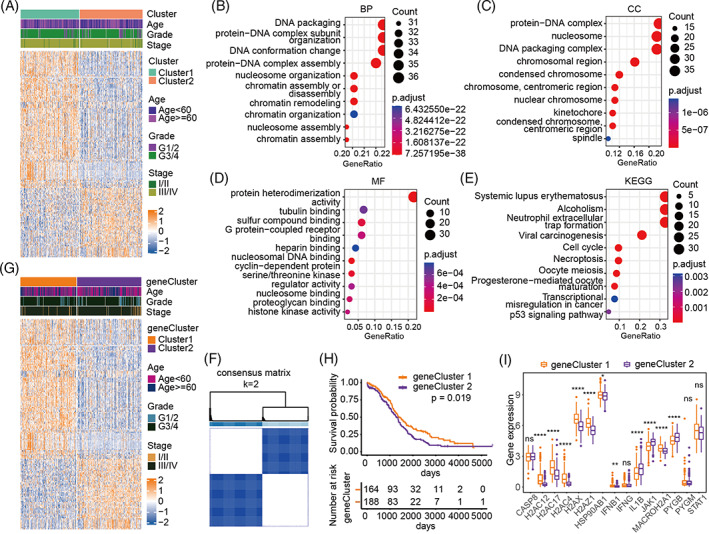
Differential expression analysis and regulatory pattern analysis of different subtypes based on necroptosis. (A) Heat map of DEG expression distribution in different subtypes. (B) Biological processes (BP) enriched in GO analysis. (C) Cellular components (CC) enriched in GO analysis. (D) Molecular functions (MF) enriched in GO analysis. (E) KEGG pathway enrichment analysis. (F) Results of unsupervised clustering analysis of DEGs (classification number *κ* = 2). (G) Heat map of DEGs in different genomic subtypes. (H) Survival status of different genomic subtypes. (I) Expression of 15 core necroptosis‐related genes associated with prognosis in different genomic subtypes.

To further investigate the different biological behaviors of each subtype, DEGs between subtypes based on the core genes were extracted using the “limma” package (Figure 3A), and the enrichment analysis of these genes was performed using the R “clusterprofiler” package. GO analysis revealed that the enriched biological processes included DNA packaging, protein‐DNA complex subunit organization or assembly, DNA conformational changes, nucleosome organization, chromatin assembly or disassembly, and chromatin remodeling (Figure 3B). Meanwhile, enriched cellular components included the protein‐DNA complex, nucleosome, DNA packaging complex, chromosomal region, condensed chromosome, and kinetochore (Figure 3C). In addition, the molecular function annotations included protein heterodimerization activity, tubulin binding, sulfur compound binding, G protein‐coupled receptor binding, and nucleosomal DNA binding (Figure 3D). KEGG pathway analysis revealed 10 distinct pathways, including systemic lupus erythematosus, alcoholism, neutrophil extracellular trap formation, viral carcinogenesis, cell cycle, necroptosis, oocyte meiosis, progesterone‐mediated oocyte maturation, transcriptional dysregulation in cancer, and p53 (Figure 3E). To further explore the patterns of necroptosis regulation, unsupervised clustering analysis was conducted, and then we classified patients into different genomic subtypes (Figure 3F). Consistent with the clustering performed using core necroptosis genes, unsupervised clustering analysis revealed two distinct genomic phenotypes, namely geneCluster1/2 (Figure 3G). Notably, geneCluster2 was also associated with a poor prognosis (Figure 3H), and most of the 15 genes showing prognostic values were differentially expressed between the two genomic subtypes (Figure 3I). Overall, the two subtypes of ovarian cancer had different expression patterns.

### 3.4 Construction and validation of a scoring system associated with necroptosis

**FIGURE 4:**
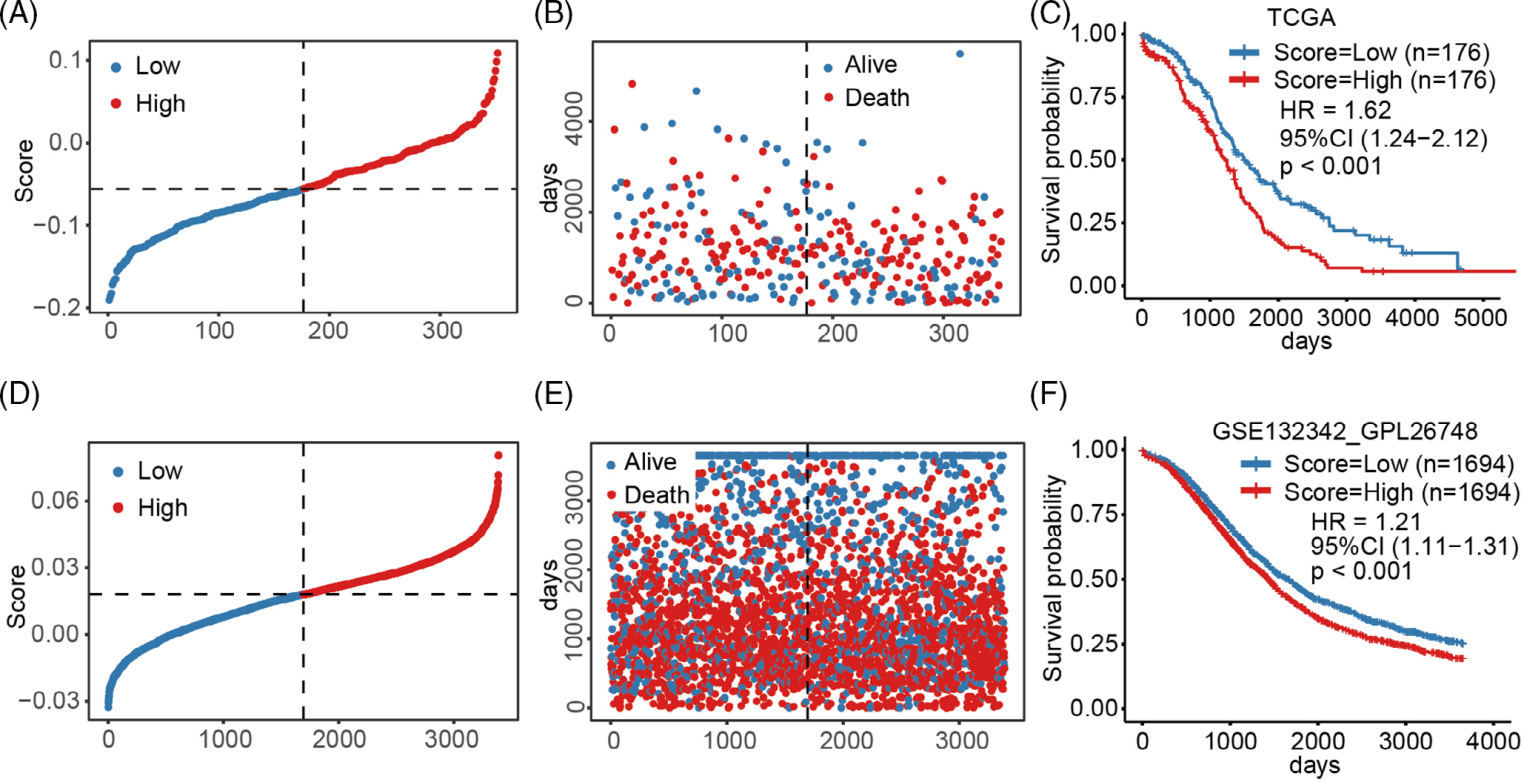
Construction and validation of a scoring system associated with necroptosis. (A) NSS distribution for each sample in the TCGA‐OV cohort. (B) Survival status of each sample. (C) Survival analysis of high‐ or low‐NSS patients. (D) NSS distribution for each sample in the GSE132342 cohort. (E) Survival status of each sample. (F) Survival analysis of high‐ or low‐NSS patients.

To quantify necroptosis for individual patients, a scoring system associated with necroptosis, namely NSS, was established. Univariate Cox regression analysis identified 51 prognosis‐related genes. Principal components 1 and 2 of the PCA method were chosen as the feature scores to construct the NSS. According to the median NSS values, samples were divided into different NSS groups with a high or low score (Figure 4A, Figure S1). The low NSS group tended to have a better survival prognosis than the high NSS group (Figure 4B,C). To verify the stability of the prognostic model, the GSE132342 cohort was analyzed using the NSS. The survival status was also significantly different between different groups, consistent with the trend observed in the training cohort. Low‐score patients tended to have better survival than those with a high NSS score (Figure 4D–F). Altogether, the NSS was established based on the DEGs between different subtypes of ovarian cancer.

### 3.5 NSS was closely associated with the clinical characteristics of ovarian cancer patients

**FIGURE 5:**
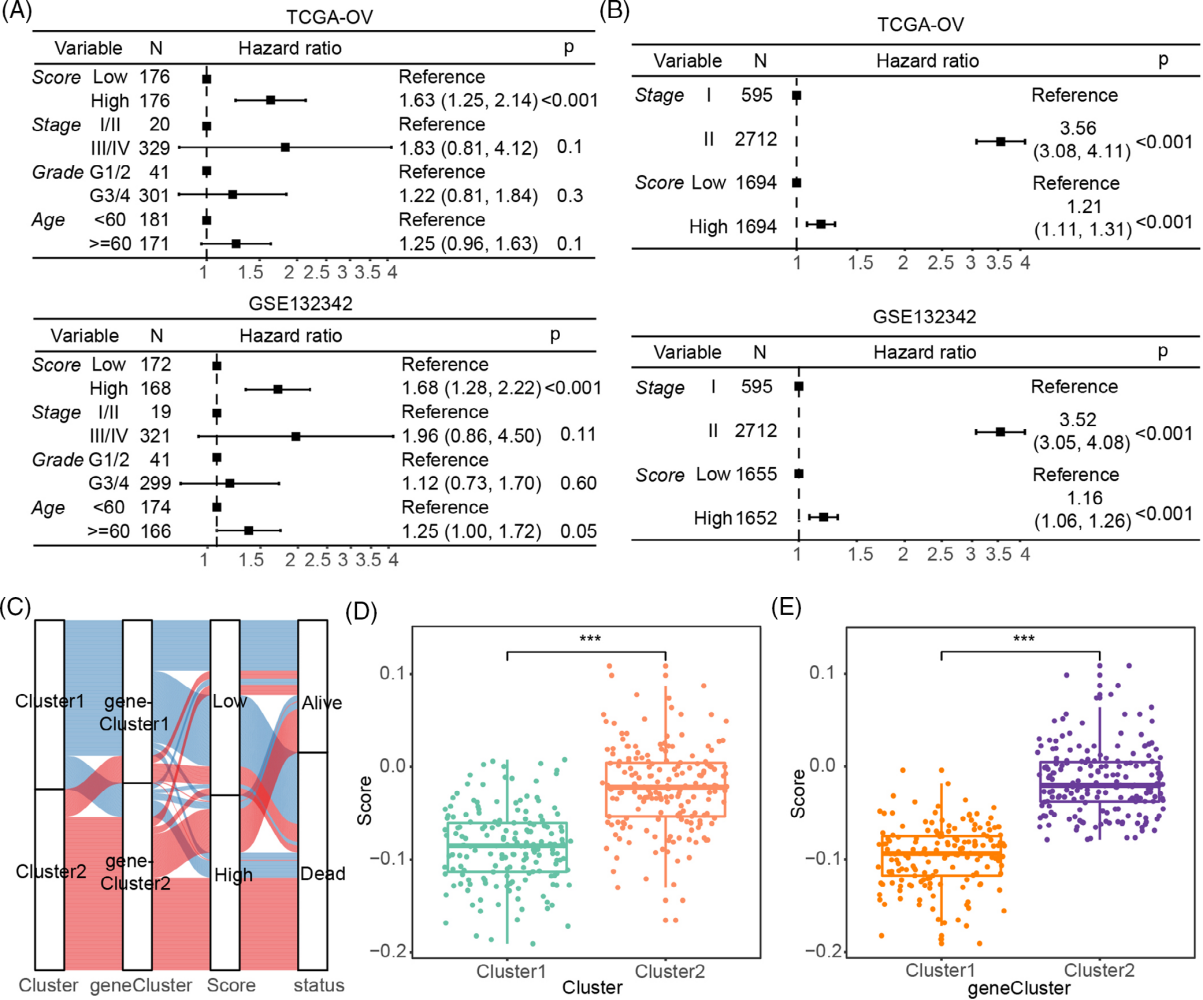
Association of NSS with clinical characteristics of patients with ovarian cancer. (A) Univariate Cox regression analysis of independent risk factors for overall survival in patients with ovarian cancer. (B) Multivariate Cox regression analysis of independent risk factors. (C) Alluvial map showing changes among cluster, geneCluster, survival status, and NSS. (D) NSS comparison in different clusters. (E) NSS comparison in different geneClusters.

Univariate Cox regression analysis indicated that the NSS had a close correlation with overall survival (OS; Figure 5A). Next, multivariate Cox regression analysis revealed that the NSS could be an independent predictor of OS (Figure 5B). An alluvial map was used to visualize the variation in individual patient attributes for different groupings based on clusters, gene clusters, NSS values, and survival status (Figure 5C). The NSS was significantly higher for the Cluster2 and geneCluster2 subtypes than for the Cluster1 and geneCluster1 subtypes (Figure 5D,E); however, there was no significant difference in the NSS among different clinical groups (Figure S3). In general, the scoring system was closely associated with the clinical characteristics of patients with ovarian cancer.

### 3.6 Exploration of molecular background based on the NSS

**FIGURE 6:**
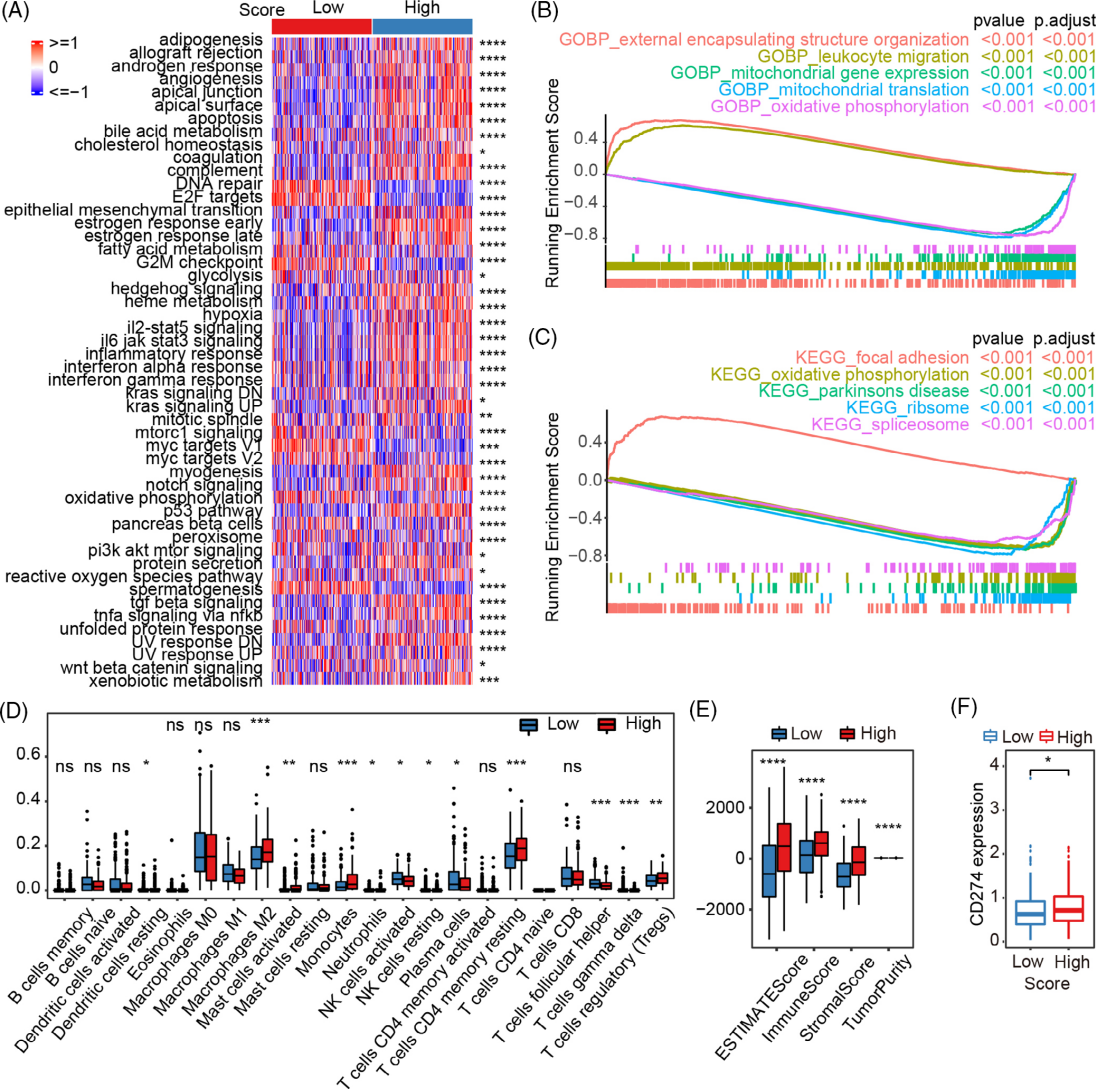
Exploration of molecular background based on the NSS system. (A) Pathways between groups with high or low NSS values. (B) Biological processes with significant changes between high‐ and low‐NSS groups. (C) KEGG pathways with significant changes between high‐ and low‐NSS groups. (D) Immune infiltration scores (cibersort) of high‐ and low‐NSS groups. (E) Comparison of ESTIMATE score, immunoscore, stromal score, and tumor purity between the two subtypes. (F) Expression of CD274 between the two subtypes.

To understand differences in the molecular background of ovarian cancer based on the NSS, single sample GSEA (ssGSEA) was performed to analyze changes in HALLMARK pathways in the TCGA‐OV cohort. Forty‐two of the fifty pathways showed significant differences between groups with high or low NSS values (Figure 6A). The NSS correlated positively with the “apical junction” and “UV response DN” pathways and negatively with the “DNA repair” and “E2F targets” pathways (Figure S4). GSEA based on gene sets from biological processes in the GO dataset (GO‐BP) revealed that the five most significant pathways were involved in external encapsulating structure organization, leukocyte migration, mitochondrial gene expression, mitochondrial translation, and oxidative phosphorylation (Figure 6B). Focal adhesion, oxidative phosphorylation, Parkinson's disease, ribosomes, and spliceosomes were identified through GSEA of KEGG pathways between different NSS groups (Figure 6C). Using CIBERSORT, we found that the high NSS group showed a higher abundance of immune cell subsets, such as M2 macrophages, monocytes, and CD4 memory resting T cells, than the low NSS group (Figure 6D). Further analysis revealed that the ESTIMATE, immune, and matrix scores of patients with a high NSS were significantly higher than those of patients with a low NSS (Figure 6E). In addition, the expression level of the immune checkpoint molecule CD274 was higher in patients with a high NSS than in those with a low NSS (Figure 6F). Overall, the NSS represents the activation of specific oncogenic pathways and immunomodulatory molecules.

### 3.7 Potential influence of the NSS as a therapeutic strategy

**FIGURE 7:**
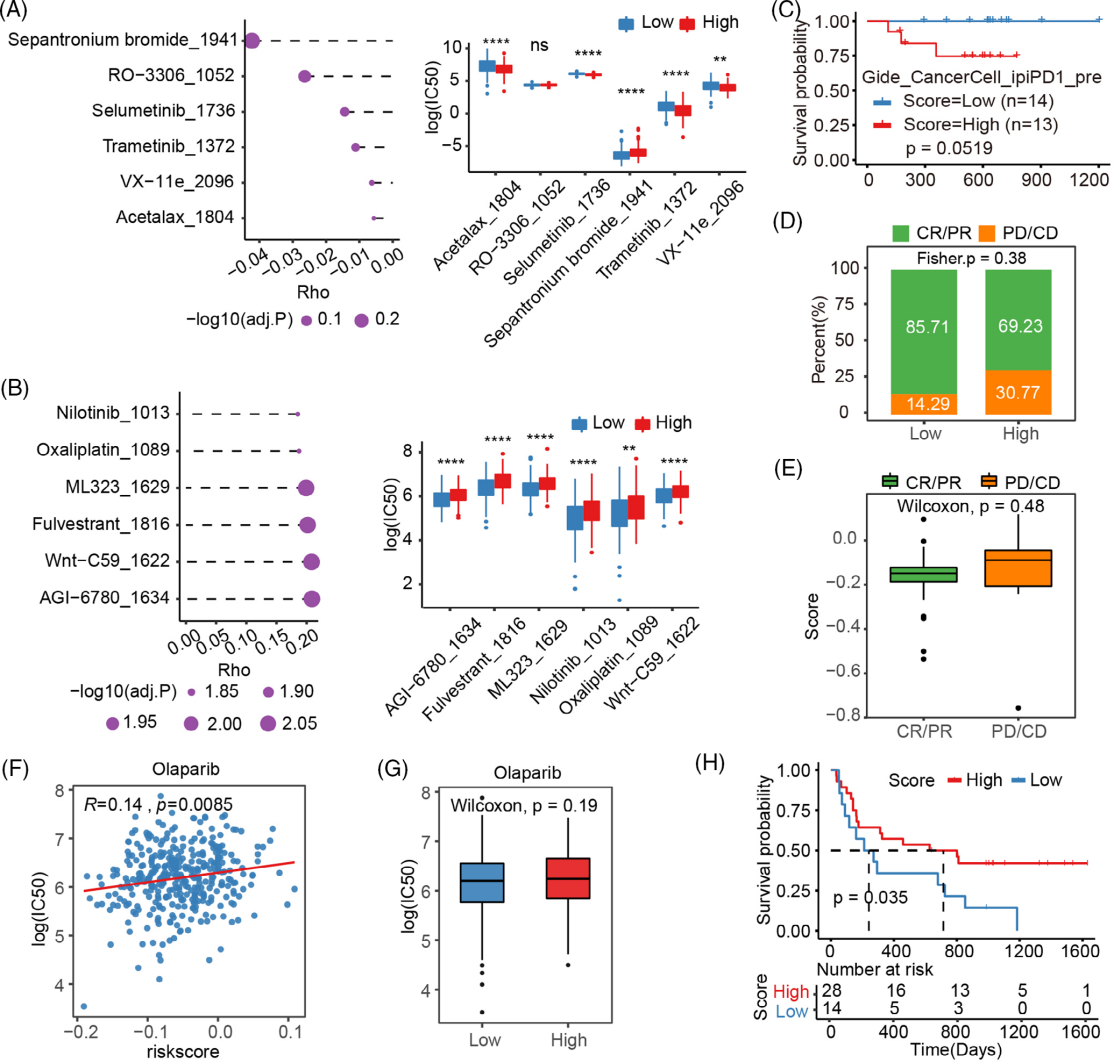
Potential influence of the NSS as a therapeutic strategy. (A) Left: NSS correlated negatively with drug log2(IC50). The top six with the highest absolute spearman correlation coefficients are shown. Right: Differences in log2(IC50) between high‐ and low‐NSS groups for the six drugs. (B) Left: NSS correlated positively with drug log2(IC50). The top six with the highest absolute spearman correlation coefficients are shown. Right: Differences in log2(IC50) between high‐ and low‐NSS groups for the six drugs. (C) Survival of high‐ and low‐NSS groups in the immunotherapy cohort. (D) Proportion of patients who did and did not respond to immunotherapy in the high‐ and low‐NSS groups. (E) Score comparison of patients with and without treatment response. (F) Correlation between olaparib IC50 and NSS. (G) Difference in olaparib IC50 between high‐ and low‐NSS groups. (H) Survival of high and low‐NSS groups in the immunotherapy cohort.

Using the R “oncoPredict” package and anticancer drug responses available in the Genomics of Drug Sensitivity in Cancer (GDSC), we combined transcriptomic data with predicted IC_50_ values for various drugs in the TCGA‐OV samples. Spearman's correlation analysis between the NSS and log2(IC_50_) of drugs suggested that drugs, such as sepatronium bromide and selumetinib, had a negative correlation with the NSS, whereas drugs, such as nilotinib and oxaliplatin, showed a positive correlation with the NSS (Figure 7A,B). These drugs included various small molecule inhibitors, including MEK/ERK pathway inhibitors (selumetinib, trametinib, and VX‐11e), a survivin inhibitor (sepantronium bromide), a CDK1 inhibitor (RO‐3306), a BCR‐ABL inhibitor (nilotinib), a USP1‐UAF1 inhibitor (ML 323), an estrogen receptor antagonist (fulvestrant), a PORCN/Wnt inhibitor (Wnt‐C59), an IDH inhibitor (AGI‐6780), and cisplatin sensitivity (oxaliplatin; Figure 7A,B). We then explored whether the NSS could predict patients who could benefit from immunotherapy using the Gide_CancerCell_ipiPD1_Pre cohort. Although no significant differences in OS were observed between these two groups following KM analysis, the low NSS group presented a trend to have a better survival (Figure 7C). No significant differences in NSS were noted between the nonresponsive (PD/CD) and responsive (CR/PR) immunotherapy groups (Figure 7D); however, the responsive immunotherapy group had a lower NSS than did the nonresponsive immunotherapy group (Figure 7E). Besides, the NSS values correlated with the IC_50_ of olaparib (Figure 7F), and samples with a high NSS tended to have a high log (IC_50_) value (Figure 7G). In the VanAllen_Science cohort with ipilimumab treatment for melanoma, patients with a high NSS were reported to benefit from treatment and had an increased survival time (Figure 7H). Therefore, the NSS has guiding significance for the prediction of therapeutic drug efficacy and is conducive for decision‐making for treatment programs.

## 4 DISCUSSION

Ovarian cancer is a common gynecological cancer that is difficult to treat. The overall survival rate remains low as there is currently a lack of effective biomarkers for predicting the prognosis or outcomes of patients. In this study, we used published transcriptomic data derived from a large ovarian cancer sample set to establish a molecular subtyping model of the core genes involved in necroptosis in ovarian cancer.

Previous evidence has suggested that necroptosis is essential in tumorigenesis, tumor progression, and tumor immunity; however, the overall effect of necroptosis in cancer has not yet been determined and may depend on the cancer type and disease stage. For instance, necroptosis was detected in areas of tumor necrosis and is thought to play a critical role in tumor growth and metastasis.^13^, ^14^ In contrast, inducing necroptosis can delay tumor progression by killing cancer stem cells or ameliorating inflammation.^15^, ^16^, ^17^ In ovarian cancer, the specific pathophysiological function of necroptosis is still unclear, and its relationship with the development of ovarian cancer is poorly studied. In this study, we performed a systematic analysis of necroptosis in ovarian cancer and found that genetic variations in necroptotic genes were frequent. We identified a series of necroptosis‐related genes involved in the pathogenesis of ovarian cancer, including *STAT1*, *CASP8*, and *IFNB1*, and provided a preliminary analysis of their interactions. GSEA demonstrated that some essential pathways are involved in necroptosis in ovarian cancer, such as DNA packaging, protein‐DNA complex assembly, chromatin remodeling and organization, functional changes in organelles, cellular adhesion, the recruitment of immune cells, and oxidative phosphorylation. These abnormally activated pathways may therefore be promising targets for drug intervention research.

Analyzing the expression of necroptotic genes in large samples to establish prognosis‐related gene signatures is important for the identification of regulatory patterns, molecular typing of tumors, guiding therapeutic options, and predicting therapeutic effects. For example, a novel necroptosis‐related gene signature established in gastric cancer was able to predict early diagnosis, prognosis, and the response to immunotherapy.^18^ Moreover, a necroptosis‐related signature was established in hepatocellular carcinoma to evaluate prognosis and profile the tumor immune microenvironment.^19^ In osteosarcoma, a necroptosis‐related gene signature was constructed and found to be strongly associated with immunity.^20^ Further, a risk model consisting of five genes (*UBD*, *ISG20*, *CXCL11*, *HLA‐DOB*, and *ATP1A3*) was constructed using least absolute shrinkage and selection operator regression and could predict the survival and prognosis of patients with ovarian cancer, while also revealing the immune microenvironment to a certain extent.^21^ In this study, we established models using two consistent clustering methods. We established molecular subtypes based on core necroptosis‐related genes and then developed genomic phenotypes of necroptosis based on unsupervised clustering analysis of 160 DEGs. The two clustering analyses yielded consistent results, suggesting that there are two different modes of necroptosis in ovarian cancer. PCA was used for dimension reduction to establish prognosis‐related gene signatures to preserve the information in the original data to the greatest extent. The different subtypes showed striking differences in survival status, immune infiltration, stromal abundance, and activated pathways. Finally, we constructed a novel scoring system named “NSS,” and validated the system using the GSE132342 cohort. The NSS had a close correlation with clinical characteristics, composition of immune cells or stroma, and activation of essential pathways, such as the apical junction, UV response, DNA repair, and E2F target pathways. In addition, a high NSS was closely correlated with higher CD274 expression, dissatisfactory response to PAPRi, poorer survival status, and poor immunotherapy sensitivity.

The close interaction between necroptosis and immune regulation has made necroptosis‐mediated anti‐tumor immune response the focus of research in recent years, although its specific regulatory mechanisms remain unclear. Necroptosis can induce CD8^+^ T cell cross‐priming, dendritic cell maturation, cytotoxic T cell cross‐priming, and IFN‐γ production in response to tumor antigen stimulation, which play essential roles in antitumor immunity.^22^, ^23^ In pancreatic ductal adenocarcinoma, the necroptotic pathway can remodel the immunosuppressive tumor microenvironment via CXCL1 and Mincle signaling, or switch tumor‐associated macrophages toward an M2‐like phenotype, which promotes immune tolerance and immunotherapeutic resistance and induces disease progression.^24^, ^25^ Overall, it was probable that chronic spontaneous necroptosis promoted tumor development by suppressing antitumor immunity, and massive induction of necroptosis could substantially undermine tumor growth and trigger immunogenic responses. The NSS established in this study correlated significantly with prognosis and could be utilized as an independent predictor of prognosis. The NSS correlated significantly with different tumor cell regulatory patterns, immune cell composition, and immune checkpoint molecule expression. Furthermore, the NSS was closely related to the sensitivity of various small molecule inhibitors. In addition, the NSS also correlated significantly with prognosis in cohorts treated with anti‐PD1 antibodies and was closely related to therapeutic responses. Furthermore, the NSS was significantly associated with drug sensitivity to olaparib and clinical outcomes in the cohort treated with anti‐CTLA4 antibodies. The NSS score can indicate the different biological composition and immune infiltration patterns in the pathogenesis of the tumor. It is closely related to small molecule inhibitors and immunotherapy, suggesting that the NSS has important application value in treatment selection, efficacy monitoring, and prognosis prediction.

Despite these notable findings, our study has some limitations. First, we were unable to validate the key necroptosis‐related genes in clinical samples of ovarian cancer. Second, the specific biological functions associated with necroptosis require further exploration and verification using in vivo and in vitro experiments. Third, the sample size of the cohort used to judge the efficacy of immunotherapy and sensitivity of PARP inhibitors was small. Therefore, it is necessary to conduct validation studies using a larger sample size to verify the utility of the NSS for selecting novel therapies or combination therapies.

## AUTHOR CONTRIBUTIONS

*Conceptualization*: Shuqin Chen and Huiling Lai; *Methodology*: Guofen Yang; *Software*: Yunyun Guo and Linxiang Wu.; *Validation*: Aligu Yusufu; *Formal analysis*: Qiyu Zhong, Zhouzhou Liao and Jianyu Ma; *Writing—original draft preparation*, Huiling Lai and Wen Shi; *Writing—review and editing*: Huiling Lai and Shuqin Chen. All authors have read and agreed to the published version of the manuscript.

## CONFLICT OF INTEREST STATEMENT

The authors declare no conflicts of interest.

## ETHICS STATEMENT

This research does not include any human or animal studies or studies that require the approval of an institutional review board.

## Supporting information


**FIGURE S1.** ROC curves for NSS in the training and validation sets.

**FIGURE S2.** Comparison of immune cell infiltration scores in the two subtypes.

**FIGURE S3.** NSS differences for different clinical characteristics.

**FIGURE S4.** Correlation between NSS and different HALLMARK pathways.

## Data Availability

Data sharing is not applicable to this article as no new data were created or analyzed in this study.

## References

[1] Sung H , Ferlay J , Siegel RL , et al. Global cancer statistics 2020: GLOBOCAN estimates of incidence and mortality worldwide for 36 cancers in 185 countries. CA. 2021;71(3):209‐249. doi:10.3322/caac.21660 33538338

[2] Matulonis UA , Sood AK , Fallowfield L , Howitt BE , Sehouli J , Karlan BY . Ovarian cancer. Nat Rev Dis Primers. 2016;2(1):1‐22. doi:10.1038/nrdp.2016.61 PMC7290868 27558151

[3] Baert T , Ferrero A , Sehouli J , et al. The systemic treatment of recurrent ovarian cancer revisited. Ann Oncol. 2021;32(6):710‐725. doi:10.1016/j.annonc.2021.02.015 33675937

[4] Dias MP , Moser SC , Ganesan S , Jonkers J . Understanding and overcoming resistance to PARP inhibitors in cancer therapy. Nat Rev Clin Oncol. 2021;18(12):773‐791. doi:10.1038/s41571-021-00532-x 34285417

[5] Kuroki L , Guntupalli SR . Treatment of epithelial ovarian cancer. BMJ. 2020;371:m3773. doi:10.1136/bmj.m3773 33168565

[6] Wu Q , Qian W , Sun X , Jiang S . Small‐molecule inhibitors, immune checkpoint inhibitors, and more: FDA‐approved novel therapeutic drugs for solid tumors from 1991 to 2021. J Hematol Oncol. 2022;15(1):143. doi:10.1186/s13045-022-01362-9 36209184 PMC9548212

[7] Wang Y , Zhang H , Liu C , et al. Immune checkpoint modulators in cancer immunotherapy: recent advances and emerging concepts. J Hematol Oncol. 2022;15:1‐53. doi:10.1186/s13045-022-01325-0 35978433 PMC9386972

[8] Tong X , Tang R , Xiao M , et al. Targeting cell death pathways for cancer therapy: recent developments in necroptosis, pyroptosis, ferroptosis, and cuproptosis research. J Hematol Oncol. 2022;15(1):1‐32. doi:10.1186/s13045-022-01392-3 36482419 PMC9733270

[9] Tang R , Xu J , Zhang B , et al. Ferroptosis, necroptosis, and pyroptosis in anticancer immunity. J Hematol Oncol. 2020;13:1‐8. doi:10.1186/s13045-020-00946-7 32778143 PMC7418434

[10] Horne CR , Samson AL , Murphy JM . The web of death: the expanding complexity of necroptotic signaling. Trends Cell Biol. 2022;33:162‐174. doi:10.1016/j.tcb.2022.05.008 35750616

[11] Galluzzi L , Kepp O , Chan FK , Kroemer G . Necroptosis: mechanisms and relevance to disease. Annu Rev Pathol. 2017;12:103‐130. doi:10.1146/annurev-pathol-052016-100247 27959630 PMC5786374

[12] Gong Y , Fan Z , Luo G , et al. The role of necroptosis in cancer biology and therapy. Mol Cancer. 2019;18(1):1‐7. doi:10.1186/s12943-019-1029-8 31122251 PMC6532150

[13] Strilic B , Yang L , Albarrán‐Juárez J , et al. Tumour‐cell‐induced endothelial cell necroptosis via death receptor 6 promotes metastasis. Nature. 2016;536(7615):215‐218. doi:10.1038/nature19076 27487218

[14] Jiao D , Cai Z , Choksi S , et al. Necroptosis of tumor cells leads to tumor necrosis and promotes tumor metastasis. Cell Res. 2018;28(8):868‐870. doi:10.1038/s41422-018-0058-y 29941926 PMC6082890

[15] Chefetz I , Grimley E , Yang K , et al. A pan‐ALDH1A inhibitor induces necroptosis in ovarian cancer stem‐like cells. Cell Rep. 2019;26(11):3061‐3075. doi:10.1016/j.celrep.2019.02.032 30865894 PMC7061440

[16] Wu NY , Huang HS , Chao TH , et al. Progesterone prevents high‐grade serous ovarian cancer by inducing necroptosis of p53‐defective fallopian tube epithelial cells. Cell Rep. 2017;18(11):2557‐2565. doi:10.1016/j.celrep.2017.02.049 28297660

[17] Guo D , Zhang W , Yang H , et al. Celastrol induces necroptosis and ameliorates inflammation via targeting biglycan in human gastric carcinoma. Int J Mol Sci. 2019;20(22):5716. doi:10.3390/ijms20225716 31739592 PMC6888087

[18] Zhou X , Zhang B , Zheng G , et al. Novel necroptosis‐related gene signature for predicting early diagnosis and prognosis and immunotherapy of gastric cancer. Cancer. 2022;14(16):3891. doi:10.3390/cancers14163891 PMC9405737 36010886

[19] Lu J , Yu C , Bao Q , Zhang X , Wang J . Identification and analysis of necroptosis‐associated signatures for prognostic and immune microenvironment evaluation in hepatocellular carcinoma. Front Immunol. 2022;13:973649. doi:10.3389/fimmu.2022.973649 36081504 PMC9445885

[20] Hua L , Lei P , Hu Y . Construction and validation model of necroptosis‐related gene signature associates with immunity for osteosarcoma patients. Sci Rep. 2022;12(1):15893. doi:10.1038/s41598-022-20217-4 36151259 PMC9508147

[21] Wang Z , Chen G , Dai F , Liu S , Hu W , Cheng Y . Identification and verification of necroptosis‐related gene signature with prognosis and tumor immune microenvironment in ovarian cancer. Front Immunol. 2022;13:894718. doi:10.3389/fimmu.2022.894718 35812403 PMC9265217

[22] Yatim N , Jusforgues‐Saklani H , Orozco S , et al. RIPK1 and NF‐κB signaling in dying cells determines cross‐priming of CD8+ T cells. Science. 2015;350(6258):328‐334. doi:10.1126/science.aad0395 26405229 PMC4651449

[23] Aaes TL , Kaczmarek A , Delvaeye T , et al. Vaccination with necroptotic cancer cells induces efficient anti‐tumor immunity. Cell Rep. 2016;15(2):274‐287. doi:10.1016/j.celrep.2016.03.037 27050509

[24] Seifert L , Werba G , Tiwari S , et al. The necrosome promotes pancreatic oncogenesis via CXCL1 and Mincle‐induced immune suppression. Nature. 2016;532(7598):245‐249. doi:10.1038/nature17403 27049944 PMC4833566

[25] Wang W , Marinis JM , Beal AM , et al. RIP1 kinase drives macrophage‐mediated adaptive immune tolerance in pancreatic cancer. Cancer Cell. 2018;34(5):757‐774. doi:10.1016/j.ccell.2018.10.006 30423296 PMC6836726

