# Supplementary figures and images for: Necroptosis‐related regulatory pattern and scoring system for predicting therapeutic efficacy and prognosis in ovarian cancer
Source: Cancer Rep (Hoboken). 2023 Sep 8;6(10):e1893. doi: 10.1002/cnr2.1893 (PMC10598257; doi:10.1002/cnr2.1893)

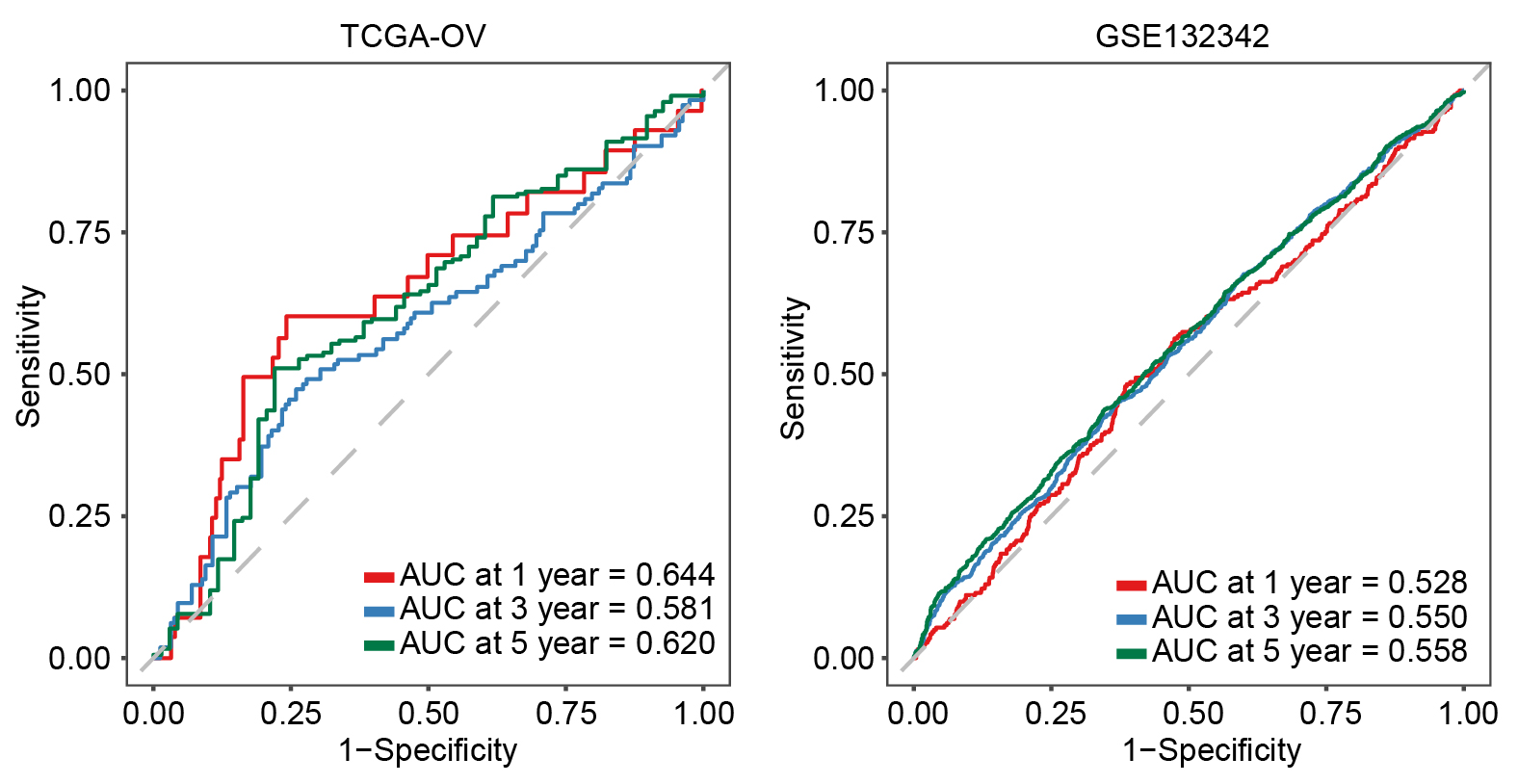

Supplement: Supplementary file 1 — FIGURE S1. ROC curves for NSS in the training and validation sets. [file CNR2-6-e1893-s001.jpg]

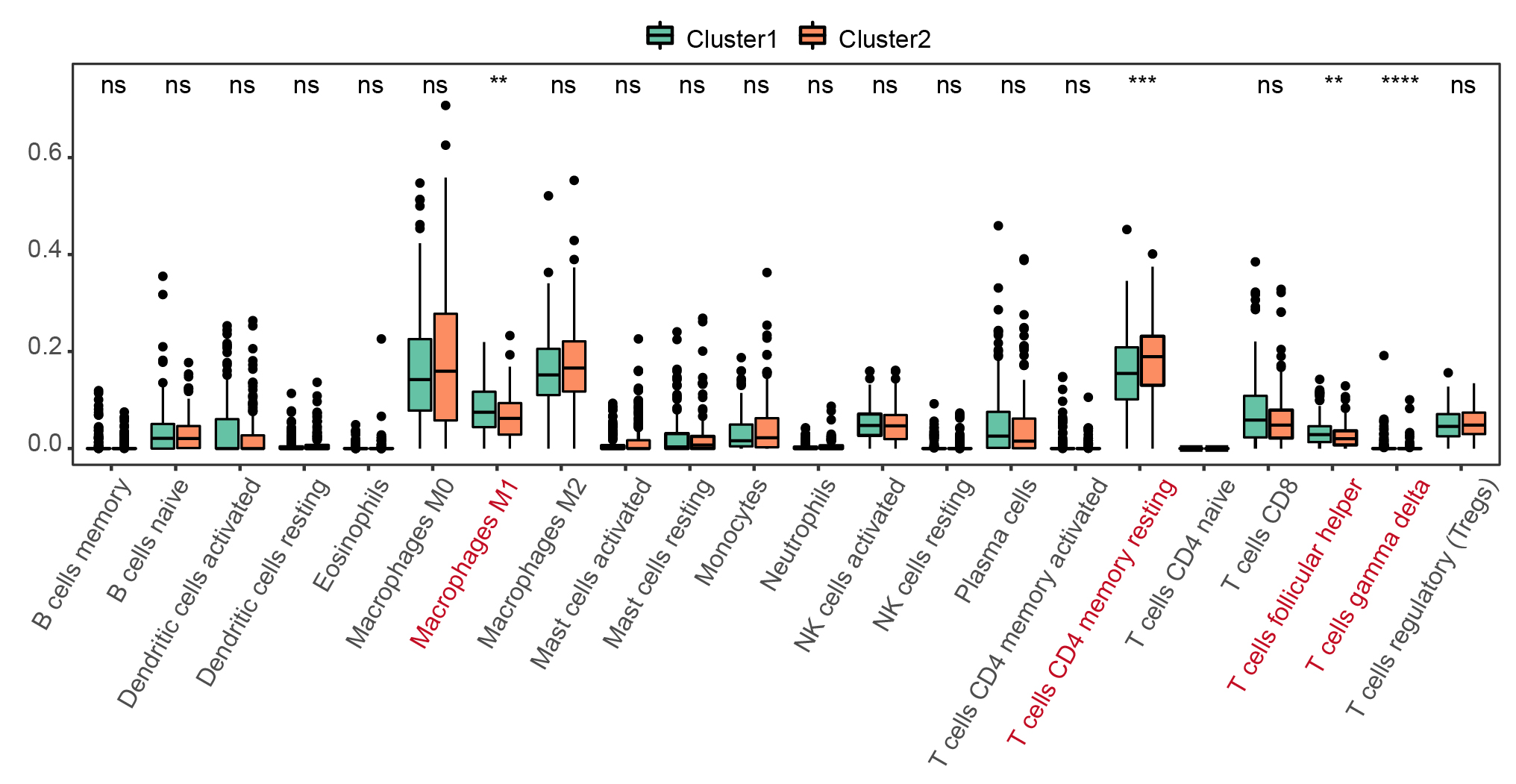

Supplement: Supplementary file 2 — FIGURE S2. Comparison of immune cell infiltration scores in the two subtypes. [file CNR2-6-e1893-s003.jpg]

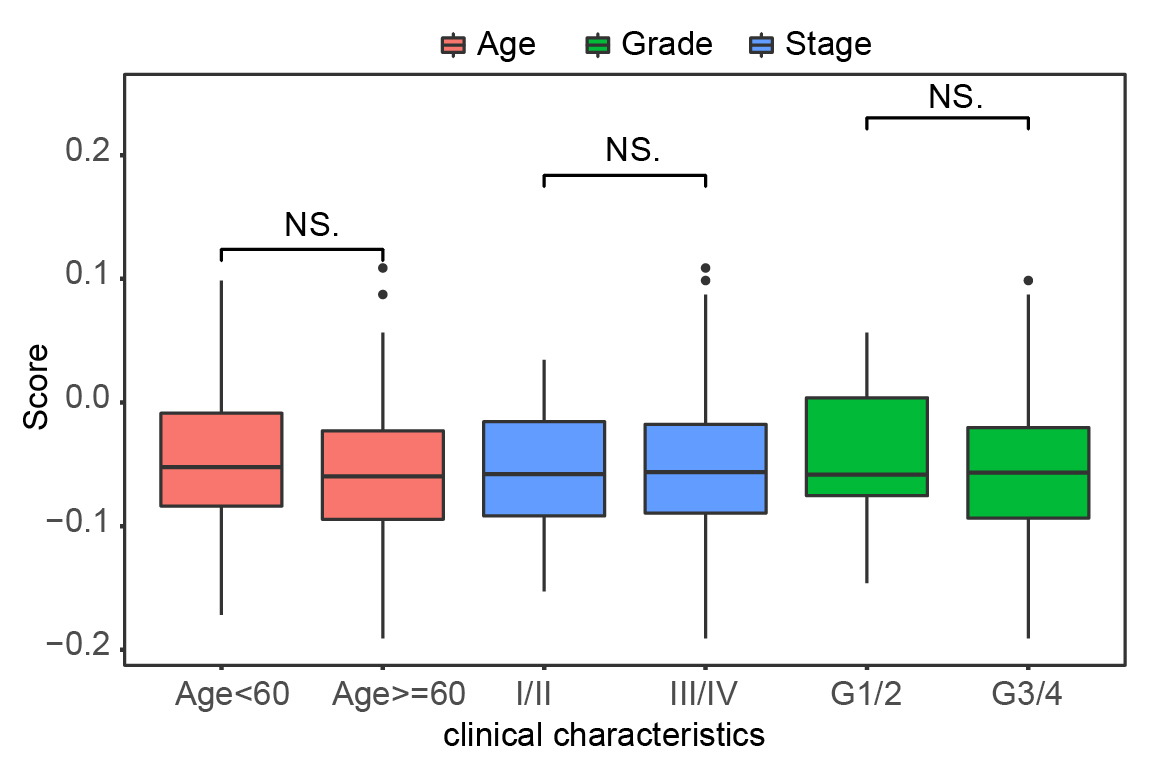

Supplement: Supplementary file 3 — FIGURE S3. NSS differences for different clinical characteristics. [file CNR2-6-e1893-s002.jpg]

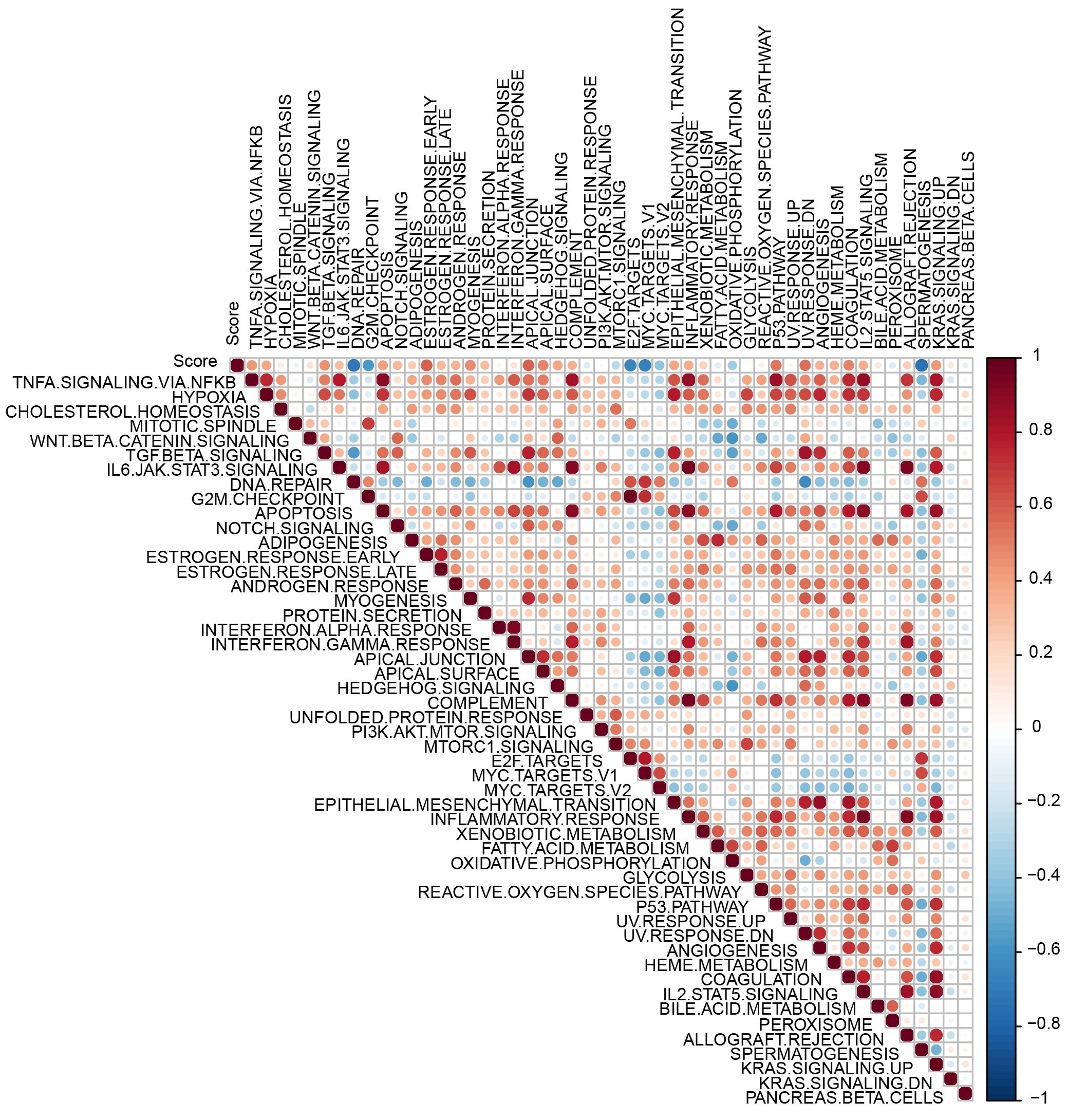

Supplement: Supplementary file 4 — FIGURE S4. Correlation between NSS and different HALLMARK pathways. [file CNR2-6-e1893-s004.jpg]
